# Problems and Future Approaches for Assessment of Periodontal Disease

**DOI:** 10.3389/fpubh.2014.00054

**Published:** 2014-05-28

**Authors:** Toshihiro Ansai, Shuji Awano, Inho Soh

**Affiliations:** ^1^Division of Community Oral Health Development, Kyushu Dental University, Kitakyushu, Japan

**Keywords:** periodontal disease, probing, bleeding from gingiva, questionnaire, epidemiology

Clinical diagnosis of periodontal disease is generally obtained by probing or attachment loss. Those have also been used in epidemiological investigations, though several problems have been pointed out, such as follows: infectious aggression by the dental instrument, time-consuming for both dental professionals and patients, and patient discomfort (e.g., pain or itching during probing).

For standardization of assessment of periodontal disease, calibration of probing between inter-examiners is needed. A systematic review of the periodontal literature has been conducted to assess the extent of reporting of calibration of probing. Searches of four electronic databases (Medline, Embase/BIDS, PubMed, and Cochrane Library) were undertaken. Search constraints were: 1996–2009; clinical trials; human studies; English language. Also, hand searches of 12 periodontal/dental journals were undertaken for 14-year period. Then, a total of 1037 papers from 668 citations as the electronic search and 369 citations as the hand search were reviewed. Of those papers, only 28% reported examiner alignment for that assessment, while 42% gave no information on examiner alignment and assessment (1). Furthermore, in 288 papers that reported the statistical methods for determining examiner alignment and assessment, approximately 20% noted kappa value, a standard index used to show whether an adequate agreement between examiners was guaranteed (>0.8 better), while no description regarding such statistics were found in 66% of those articles (1).

Although it is important to begin by considering the definition of periodontal disease, no uniform case definition exists. Gingival inflammation (gingivitis) (2), deepened pockets (3), periodontal bone loss (4), and a combination of different oral parameters (5) have been used to define periodontal disease. To further complicate the matter, a wide variation of cut-off values for these parameters have been utilized to identify the presence of periodontal disease, such as attachment loss greater than 3 mm per year (6), attachment loss greater than 3 mm and probing depth greater than 4 mm in at least 30% of sites measured (7), attachment loss greater than 4 mm in at least 30% of sites measured (8), and classification based on the proportion of teeth with greater than 4 mm of probing depth (9). Therefore, comparisons of results from different studies include uncertainty and it is difficult to make conclusions from meta-analyses. Thus, since the parameters used for diagnosis of periodontal disease are not straightforward, we consider that a probing method is inappropriate for determination of underlying disease status.

We would propose the following four methods as alternatives to probing for use in epidemiological studies and population-based community examination: (1) number of lost teeth, (2) bleeding from gingiva, (3) self-reported questionnaire of periodontal condition, and (4) combination of general medical assessment and non-invasive tests of the gingiva. Problems with these methods and future perspective are discussed below.

(1) Number of lost teeth: tooth loss present an accumulative burden throughout life. As noted by Tu and Gilthorpe (10), tooth loss might be a better indicator than probing as a marker of lifetime oral health, and is less prone to measurement error. Importantly, a kappa value greater than 0.9 can be obtained for calibration of assessment of tooth loss, indicating good reliability. On the other hand, it is not clear whether tooth loss is the result of periodontal disease or dental caries or both. For example, according to a dental professional survey conducted in 2005 (*n* = 2001), the main reason for tooth loss in 42% of the subjects was periodontal disease, while dental caries was noted in 33%, though gender and age differences were also found, with periodontal disease predominant in subjects over 45 years old (11). Another controversial issue is whether tooth loss itself reduces pathological condition related to periodontal disease. In other words, though tooth loss shows a past condition of inflammation, gingival inflammation related to periodontal condition may not be present.
(2) Bleeding from gingiva: the pathogenesis of periodontal disease includes existence of gingival inflammation, thus it is reasonable to assess bleeding from the gingiva. To date, gingival bleeding has been used for clinical diagnosis of periodontal disease. Recently, a test kit for simple assessment of occult blood has been developed in Japan, and found to be effective for screening of periodontal disease, with a sensitivity and specificity of 0.72 and 0.52, respectively (9). Furthermore, multivariate logistic regression analysis performed in that study showed that the results of the test were significantly associated with the proportion of teeth with gingival bleeding and with probing depth greater than 4 mm, even after adjusting for several confounding factors. The most important feature of the kit is its non-invasive design, since saliva is used as the test sample. Briefly, each subject refrained from eating and drinking from the previous night. For specimen collection, each rinsed their mouth for 10 s with 3 ml of distilled water, then spat it into a small paper cup. We think that bleeding from the gingiva would be a better indicator of present periodontal tissue condition than tooth loss.
(3) Self-administrated questionnaire: recently, self-reported means for determining periodontal disease have received attention, primarily due to their simplicity and low cost. In an assessment of the validity of self-reported questionnaires, improvements in sensitivity and specificity occurred when such questions, as “Gum surgery in the past?”, “Sore gums in the past?”, “Scaling in the past?”, “Bleeding gums now?”, “Periodontal surgery in the past 2 years?”, and “Chewing satisfaction?” were included (12). Furthermore, Eke et al. (13) reported that the combined effects of demographic measures (age, sex, race/ethnicity, education, smoking, etc.) and responses to five self-reported questions had a sensitivity of 85% and specificity of 58% for predicting periodontitis of mild or greater severity, and produced an area under the curve (ROC) of 0.81.
(4) Combination of general medical assessment and non-invasive tests for gingiva: recent studies have shown bi-directional interrelationships between periodontal disease and systemic condition including metabolic syndrome, diabetes, and obesity (14–17). Recently, Japanese researchers investigated the association between gingival bleeding as a means of screening for periodontal disease and metabolic syndrome-related factors among 488 subjects aged 40–74 years who underwent specific health checkups. The subjects were divided into two groups, screen-positive and -negative. The results of logistic regression analyses showed that the adjusted odds ratios for obesity and metabolic syndrome in the screen-positive group were 1.64 (95% confidence interval, 1.03–2.61) and 2.49 (1.34–4.63), respectively (18). Those authors concluded that there was a significant association between gingival bleeding in saliva and metabolic syndrome.

Based on those results showing an association between periodontal disease and metabolic syndrome, we propose a novel approach that utilizes a combination of general medical assessment including assessment for metabolic syndrome and simple assessment of gingival bleeding. Our concept is consistent with the common risk factor approach proposed by Sheiham and Watt (19). They noted that non-communicable disease is associated with common risk factors such as diet, smoking, stress, alcohol, physical fitness, and others, and suggested that an approach for oral health promotion should be preceded as a collaborative approach including such common risk factors, while conventional oral health approaches were neither effective nor efficient. Furthermore, in many developed countries including Japan, low participation in routine screening examinations annually performed for community-dwelling individuals remains an unsolved issue. As a result, our novel approach may provide a solution, since such medical examinations are supported by medical law of the Ministry of Health in Japan.

In this article, we examined several approaches used for periodontal assessment, including those lacking periodontal probing. We think that elimination of probing is an urgent issue in dental medicine. Furthermore, objective comparisons of nationwide and international data regarding periodontal disease would be indispensable for planning oral health strategies for general population. Therefore, we suggest construction of a novel consensus for international standardization regarding assessment of periodontal disease in the near future. In addition, standardized non-probing assessments must become standard.

## Conflict of Interest Statement

The authors declare that the research was conducted in the absence of any commercial or financial relationships that could be construed as a potential conflict of interest.
